# Comparative study of eggshell antibacterial effectivity in precocial and altricial birds using *Escherichia coli*

**DOI:** 10.1371/journal.pone.0220054

**Published:** 2019-07-24

**Authors:** Xia Chen, Xingzheng Li, Zhaoxiang He, Zhuocheng Hou, Guiyun Xu, Ning Yang, Jiangxia Zheng

**Affiliations:** National Engineering Laboratory for Animal Breeding and MOA Key Laboratory of Animal Genetics and Breeding, College of Animal Science and Technology, China Agricultural University, Beijing, China; Tokat Gaziosmanpasa University, TURKEY

## Abstract

In this study, we compared the antibacterial effectivity of the eggs of six precocial and four altricial bird species using *Escherichia coli*, based on their eggshell traits. The ultrastructure of eggshell was observed using a scanning electron microscope (SEM). According to SEM results, eggs from precocial birds (chicken, turkey, quail, duck, ostrich, and goose) had cuticle on the eggshells, while eggs from altricial birds (pigeon, budgerigar, munia, and canary) did not. The environment/selection pressure may induce the divergent evolution process in eggs of precocial and altricial birds. The *E*. *coli* experiment results showed that chicken, turkey, quail, duck, and goose eggs, with a high cuticle opacity, exhibited a much lower *E*. *coli* penetration rate. In contrast, the eggs with poor (ostrich) or without (pigeon, budgerigar, munia, and canary) cuticle exhibited a higher penetration rate. It is suggested that cuticle is a main barrier against bacterial penetration in precocial birds’ eggs. Turkey and quail eggs showed the lowest *E*. *coli* contamination rate (3.33% and 2.22%, respectively), probably because of the tightly connected nanosphere structure on their cuticle. As for altricial birds’ eggs, the eggs of budgerigar, munia, and canary with small pore diameter (0.57 to 1.22 μm) had a lower *E*. *coli* penetration rate than pigeon eggs (45.56%, 66.67%, 50%, and 97.78%, respectively, *P* < 0.05), indicating that pore diameter played a significant role in defending against bacterial trans-shell invasion. We found that eggshell thickness and pore area decreased with egg size. The cuticle quality had no relationship with egg size, but was closely related to the bird species. The *E*. *coli* penetration rate of altricial birds’ eggs was significantly higher than that of precocial birds’ eggs, mainly because the pores are exposed on the eggshell surface and cuticle protection is absent. This study provides detailed information on the eggshell cuticle, which gives insight into the cuticle evolution process that occurred in precocial and altricial bird species. Moreover, the results of *E*. *coli* penetration may help understanding the antibacterial behavior in birds.

## Introduction

Poultry eggs are important animal-protein sources for humans. However, eggs and egg products are easily contaminated by pathogenic bacteria (i.e., *Salmonella enteritidis*, *Bacillus cereus*, and *Escherichia coli*) [1]. Recently, the United States government recalled nearly 207 million potentially *Salmonella*-infected eggs [2], which posed a great threat to human health. To ensure egg products’ safety, it is quite important to understand the bacterial invasion mechanism in eggs. Eggs can be infected by bacteria through two major routes: vertical and horizontal transmission [3]. Vertical transmission points to the bacterial contamination process during egg formation in the hens’ oviduct. Horizontal transmission, also called trans-shell contamination, occurs when eggs are exposed to a contaminated environment and microorganisms penetrate the egg.

Cuticle, deposited in the shell gland pouch within hours before oviposition [4], is reported to be an effective barrier against microbial penetration [5,6]. The cuticle forms a physical barrier to bacterial penetration by covering the pores on the eggshell surface [7]. The chemical composition of eggshell cuticle also plays an important role in limiting bacterial contamination. Some antibacterial proteins (i.e., c-type lysozyme, ovotransferrin, and ovocalyxin-32) have been detected in the eggshell cuticle [8,9,10]. D'Alba et al. [11] proposed that the tightly connected nanostructure of the cuticle also contributes to the antimicrobial defense system.

Many avian eggs are protected from microbes by eggshell cuticle, including chicken, duck, goose, and quail eggs [11,12,13]. Evaluating the cuticle quality will help us understand the antibacterial effectivity of avian eggs. Leleu et al. [14] and Bain et al. [6] established two methods to evaluate the cuticle quality in chicken eggs. However, neither of these methods has been applied to evaluate the cuticle quality among eggs of different bird species that may depend on the difference in eggshell colors. Previously, we proposed an opacity method to effectively evaluate the cuticle quality among differently colored eggshells [15].

Not all bird eggs have a cuticle layer on the surface of the shell; examples of such eggs are those of budgerigar and pigeon [16,17]. Some other factors may be responsible for their antibacterial properties. The pore openings on the surface of an eggshell not only allow for gas and water exchange, but also are the pathway of bacterial trans-shell invasion [7,18]. Consequently, eggshell thickness was also considered an important factor to hamper bacterial contamination in chicken eggs [19]. However, some researchers suggested that bacterial trans-shell penetration was not related to eggshell quality [20]. A clear understanding of these factors among different bird species would be helpful for the research of egg safety, especially for eggs laid in the wild.

With improvements of our living standard, the demand for poultry eggs has expanded from traditional chicken and duck to other more “exotic” species, such as quail, turkey, pigeon, and ostrich eggs. However, compared with chicken eggs, the cuticle quality and antibacterial effectivity of other species’ eggs has been investigated less often. Therefore, the goals of this study are to investigate the influence of eggshell traits on the antibacterial effectivity by an *E*. *coli* penetration trial on ten bird species, six precocial species (chicken, turkey, quail, duck, goose, and ostrich) and four altricial ones (pigeon, budgerigar, munia, and canary). The eggshell cuticle quality of ten bird eggs was evaluated with the opacity method, and the microstructure of eggshell cuticle was also observed through scanning electron microscopy (SEM). In addition, the eggs’ eggshell thickness and pore diameter were also measured.

## Materials and methods

### Ethics statement

The animal care protocol used in the present study was approved by the Animal Welfare Committee of China Agricultural University (permit number: AW08059102-1).

### Eggs

Sampled eggs include six precocial species: Dwarf Layer (*Gallus gallus*), Bettina turkey (*Meleagris gallopavo*), Japanese quail (*Coturnix japonica*), Beijing duck (*Anas platyrhynchos*), greylag goose (*Anser anser*), and ostrich (*Struthio camelus*), and four altricial birds’ eggs: snow pigeon (*Columba leuconota*), budgerigar (*Melopsittacus undulatus*), munia (*Lonchura striata*), and canary (*Serinus canaria*).

The Experimental Unit of China Agricultural University (Beijing, China) supplied chicken eggs. The National Center of Performance Testing of Poultry (Beijing, China) supplied eggs for the remaining species. All birds used in this study were healthy and had not previously experienced antibacterial treatment. Housing, management, feeding, and husbandry conditions were consistent with the recommendations provided by the poultry companies. All birds were fed by the guidelines of the poultry companies that ensure the nutritional requirements of the individual species are met. Ninety eggs were collected per species except ostriches, for which 30 eggs were used. All eggs were collected within 24 h of laying and were tested within 48 h. Eggs were visually inspected through candling to select intact eggs (i.e., no cracks or pinholes). Then, those eggs were weighed on a small electronic scale (AL240, 0.01–210 g, Mettler Toledo, Shanghai, China) except for the ostrich eggs, which were weighed on a larger electronic counting scale (ACS-3, 10 g– 3 kg, Hua Chao, Shanghai, China). A line was drawn along the long axis of the egg with a pencil. One side was marked as *E* (*Escherichia coli* inoculation) and the other side marked as *S* (stained with MST cuticle blue, MS Technologies Ltd, UK).

### Assessment of cuticle deposition

The method for evaluating cuticle quality was described by Chen et al. [15]. Cuticle quality was evaluated based on differences in cuticle opacity before and after staining. A high alpha (α) value denoted high staining affinity, implying more cuticle deposition. Cuticle opacity was calculated using Eqs 1 to 7. Each egg was measured at three points: the blunt end, equator, and sharp end. Cuticle quality per egg was determined from the mean value of these points.

First, X, Y, and Z values of the eggshell *S* surface were obtained with a spectrophotometer (CM-2600d; Konica Minolta, Japan) using the XYZ color space systems. Subsequently, eggs were immersed in MST cuticle blue for 1 min, rinsed in clean water to remove excess stain, and placed on a plastic flat to dry for 24 h. Finally, X, Y, and Z values of stained eggshells were measured using the procedure described above.

$$R=(1.055\times {\left(\frac{3.241\times X-1.537\times Y-0.499\times Z}{100}\right)}^{0.417}-0.55)\times 255$$ (1)

$$G=(1.055\times {\left(\frac{-0.969\times X-1.876\times Y-0.042\times Z}{100}\right)}^{0.417}-0.55)\times 255$$ (2)

$$B=(1.055\times {\left(\frac{0.056\times X-0.204\times Y-1.057\times Z}{100}\right)}^{0.417}-0.55)\times 255$$ (3)

$$\alpha_{R}=1-\frac{R_{a}-R_{d}}{R_{b}-R_{d}}$$ (4)

$$\alpha_{G}=1-\frac{G_{a}-G_{d}}{G_{b}-G_{d}}$$ (5)

$$\alpha_{B}=1-\frac{B_{a}-B_{d}}{B_{b}-B_{d}}$$ (6)

$$\alpha =\frac{\alpha_{R}+\alpha_{G}+\alpha_{B}}{3}\times 100$$ (7)

where RGB is an additive color model in which red (R), green (G), and blue (B) light are added together in various ways to reproduce a broad array of colors. In the XYZ model, Y is luminance, Z is quasi-equal to blue stimulation, and X is a mix (a linear combination) of cone response curves. XYZ color space can be converted into RGB color space by the formulas above, and then converted into opacity. *α* is the cuticle opacity, subscript “a” indicates egg values post-staining, “b” indicates pre-staining values, and “d” indicates values for the cuticle blue dye.

### *Escherichia coli* inoculation

The *E*. *coli* penetration assays were conducted following previously described methods [6]. The *E*. *coli* strain HB-101 K-12 with pGLO (Bio-Rad Laboratories Co., Ltd., Shanghai, China) was grown in lysogeny broth (containing 100 μg mL^-1^ ampicillin Na and 5 mM L-arabinose; Sigma-Aldrich, Shanghai, China), and shaken overnight at 37C. Cultures were inoculated at a dilution of 1:50 in fresh lysogeny broth, then grown to an OD_600_ of approximately 0.4 at 37C. Thereafter, the broth was placed into an ice bath.

Eggs were lightly swabbed with ethanol, then incubated in a sterile, plastic egg box for 2 h at 37C. Then, the *E* side of the egg was immersed in ice-chilled broth for 10 min inoculation. Subsequently, the inoculated eggs were placed on a sterilized bench, to allow the culture to dry on the eggshell surface for about 15 min. Finally, each egg was transferred individually into a new sterile plastic bag and incubated for 24 h at 37C.

All eggs were removed from the incubator and placed on a sterile clean bench at room temperature for 2 h. Egg contents were drained through a hole of approximately 1 cm^2^, prepared with a rotary tool (Dremel, S-B Power Tool Company, Chicago, IL, USA). The egg was cut into two halves along the longitudinal axis with the rotary tool. The presence of *E*. *coli* was determined by observing the luminescent spots on the inner eggshell surface under a long-wave UV light (LUYOR-1144A, LUYOR Corporation, Shanghai, China). The number of *E*. *coli* in the egg was determined by the number of luminescent spots on the inner surface under UV light. Contaminated eggs were grouped into three levels: light (with 1–3 luminescent spots), moderate (with 4–10 luminescent spots), and severe (with more than 10 luminescent spots).

### Measurement of eggshell thickness and pore density

The eggshells were characterized by the traits of eggshell thickness, pore diameter, and pore area. Eggshell thickness and pore density were measured at the equator of eggs. Eggshell thickness was determined using a digital display micrometer gauge (Mitutoyo, Kawasaki, Japan). Pores were counted following published methods [21,22]. Eggshell fragments of chicken, turkey, quail, duck, goose, quail, pigeon, and ostrich were boiled in 1% KOH to remove the inner eggshell membrane and outer cuticle. Immersion time (~3 min for chicken, 4 min for duck and turkey, 5 min for goose, 90 s for quail and pigeon, and 8 min for ostrich) was dependent on eggshell thickness. Eggshell membranes of budgerigar, munia, and canary were too thin to boil so they were carefully removed using tweezers. Subsequently, all eggshells were rinsed in clean water for 1 min, immersed in 75% ethanol for 1 min, and then immersed in 1% HCl for 20 s to enlarge eggshell pores. Finally, eggshell fragments were rinsed in clean water for 1 min and placed on a table to dry. Eggshell inner surfaces were painted with 0.2% methylene blue (Solarbio, Beijing, China). Once the dye was dry, pores within a 0.25 cm^2^ area were counted under a microscope.

### Eggshell ultrastructure

SEM (S-3400 N and SU8010, Hitachi, Japan,) was used to observe ultrastructural features of the outer surface layer, cross-section, and cuticle layer of eggshells [23]. Ten eggs per species were used to measure cuticle layer thickness. Pore diameter and area of single pores were determined through SEM from the same eggshells as were used for pore density calculation. To make the results more reasonable and more accurate, we measured the single pore area and the short axis diameter of the pore (in case the pore shape was not a circle) for eggs of ten bird species. Five eggs per species were prepared, and for each egg three pieces were taken around the equator. Eggshell pieces were mounted on an aluminum stub and gold sputter-coated using an EIKO IB-3 (EIKO Engineering CO., Ltd, Japan) for about 15 min. Thereafter, they were viewed and photographed under the SEM.

### Statistical analysis

The pore area was measured using the software Image J. All statistical analyses were performed with the statistical software RStudio (version 3.4.0, 2017) and figures were plotted in Origin Pro 2018. Descriptive statistics and one-way ANOVA were used to analyze eggshell thickness, pore density, pore diameter, cuticle layer thickness, and cuticle opacity per species. The model used for the one-way ANOVA analysis was:

$$Y_{ij}=\mu +\tau_{i}+\epsilon_{ij}$$

where *Y*_*ij*_ represents traits (eggs weight, eggshell thickness, cuticle opacity, cuticle layer thickness, pore density, pore area, and pore diameter) analyzed in this study, *μ* represents the common effect for each test, *τ*_*i*_ represents the species-specific or bird types-specific (precocial vs altricial birds) main effect (factor), and *ϵ*_*ij*_ represents individual-specific random error. The significance level chosen for all analyses was *P* < 0.05.

## Results

### Eggshell traits

#### Cuticle opacity

Cuticle was not detected in altricial birds’ (pigeon, budgerigar, and munia) eggs (Table 1). As for precocial birds’ eggs, quail eggs had the highest cuticle opacity (52.29 ± 14.73%), while ostrich eggs had the poorest opacity (5.39 ± 3.84%). The chicken egg cuticle opacity was 25.34 ± 13.32%, which was significantly poorer than duck and turkey eggs (*P* < 0.05).

**Table 1:** Eggshell thickness, pore density, pore diameter, cuticle opacity, and cuticle layer thickness in ten bird species.

Traits	Chicken	Turkey	Quail	Duck	Goose	Ostrich	Pigeon	Budgerigar	Munia	Canary
**EW (g)**	43.27 ± 3.18^d^	81.98 ± 8.58^c^	8.77 ± 0.66^f^	76.45 ± 0.84^c^	136 ± 9.92^b^	1357 ± 82.20^a^	20.06 ± 1.03^e^	2.02 ± 0.26^f^	1.23 ± 0.19^f^	1.60 ± 0.17^f^
**α**	25.34 ± 13.32^d^	38.58 ± 7.63^c^	52.29 ± 14.73^a^	39.08 ± 8.87^c^	45.62 ± 13.89^b^	5.39 ± 3.84^e^	—	—	—	—
**CLT (μm)**	5.60 ± 2.39^c^	8.23 ± 1.02^b^	10.63 ± 2.16^a^	8.07 ± 1.40^b^	9.33 ± 2.06^b^	1.02 ± 0.98^d^	—	—	—	—
**EST (μm)**	332.60 ± 28.39^d^	366.38 ± 30.53^c^	192.18 ± 19.32^e^	366.10 ± 32.04^c^	486.94 ± 60.30^b^	1955.51 ± 95.65^a^	180.38 ± 22.72^e^	92.61 ± 11.66^f^	65.03 ± 5.01^g^	57.18 ± 5.54^g^
**SPA (μm**^**2**^**)**	414.29 ± 260.81^c^^d^	327.18 ± 168.907^d^	212.19 ± 74.24^e^	514.26 ± 258.56^c^	671.52 ± 267.19^b^	23148.14±9891.43^a^ 9891.43^a^	195.77 ± 111.56^e^	1.57 ± 0.66^f^	0.69 ± 0.30^f^	0.32 ± 0.10^f^
**PDM (μm)**	14.13 ± 7.37^b^	12.60 ± 4.05^b^	12.99 ± 2.40^b^	17.21 ± 5.48^a^	11.85 ± 4.17^b^	16.20 ± 4.58^a^	13.24 ± 5.19^b^	1.22 ± 0.28^c^	0.85 ± 0.18^c^	0.57 ± 0.10^c^
**PD (cm**^**-2**^**)**	76.10 ± 28.97^a^^b^^c^	72.10 ± 23.53^a^^c^^d^	88.30 ± 28.24^a^^b^	71.72 ± 21.48^b^^c^^d^	53.55 ± 20.53^e^	7.17 ± 2.15^f^	55.76 ± 17.64^d^^e^	76.37 ± 38.15^a^^b^^c^	88.84 ± 45.67^a^	90.84 ± 49.59^a^
***n***^**E**^	14	3	2	11	14	23	88	41	60	45
***N***	90	90	90	90	90	30	90	90	90	90
**PR (%)**	15.56	3.33	2.22	12.22	15.56	76.67	97.78	45.56	66.67	50.00

Cuticle layer thickness and pore diameter was measured under a scanning electron microscope. Single pore of ostrich egg was made up of many small pores. Its area was determined as the sum of areas of these small pores. EW, egg weight; α, cuticle opacity; CLT, cuticle layer thickness; EST, Eggshell thickness; SPA, single pore area; PDM, pore diameter; PD, pore density; *n*^E^, number of eggs penetrated by *E*. *coli*; *N*, total number of eggs; PR, penetration ratio;—, cuticle layer absence.

^a, b, c, d, e, f, g^, means on the same row with different letters are significantly different (*P* < 0.05).

#### Cuticle ultrastructure

**Fig 1:**
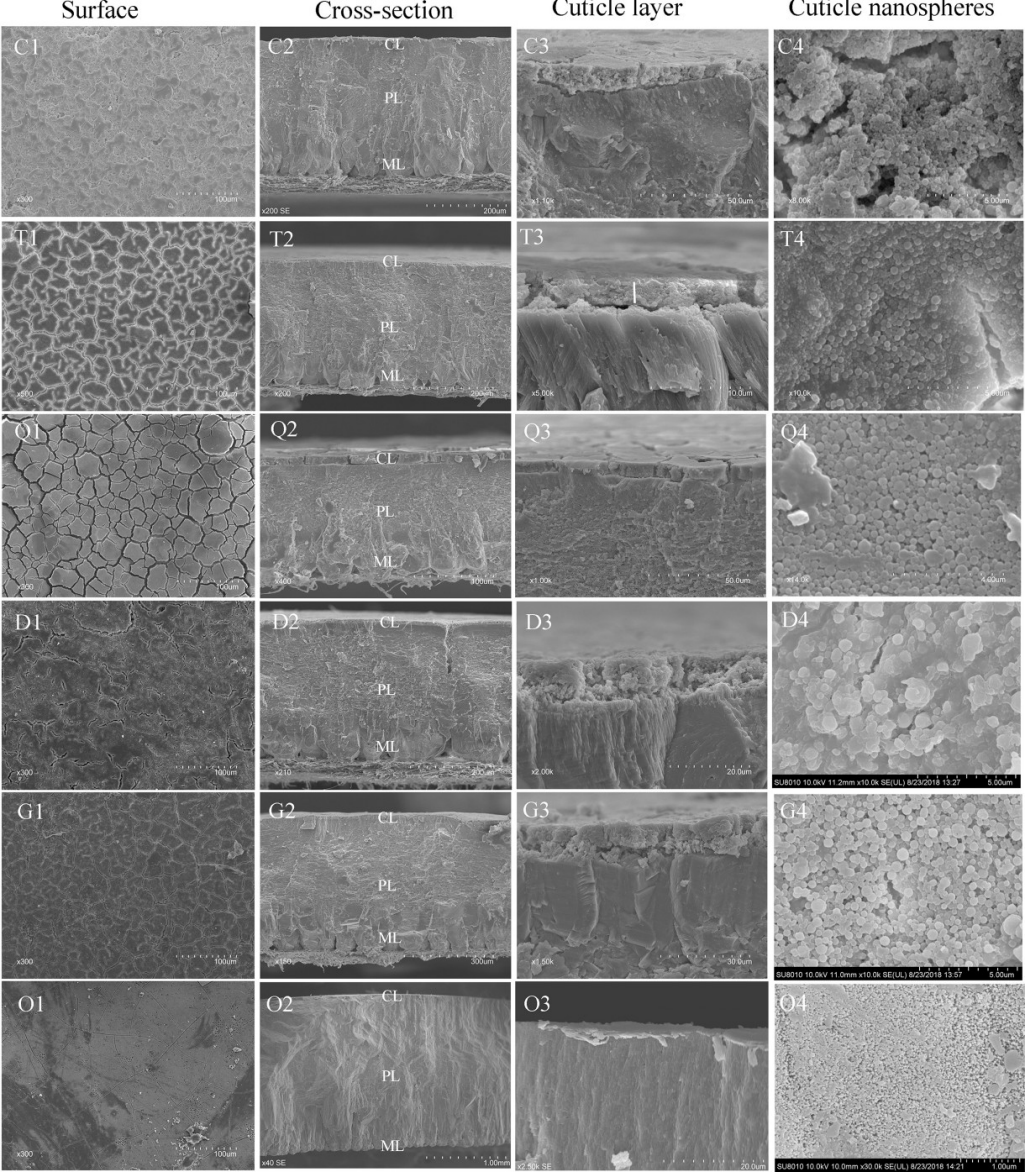
Scanning electron micrographs showing eggshell surface, cross-section, cuticle layer, and cuticle nanospheres of chicken (C), turkey (T), quail (Q), duck (D), goose (G), and ostrich (O). Cross-sections revealed that the shell was divided into a mammillary layer (ML), palisade layer (PL), and cuticle layer (CL).

**Fig 2:**
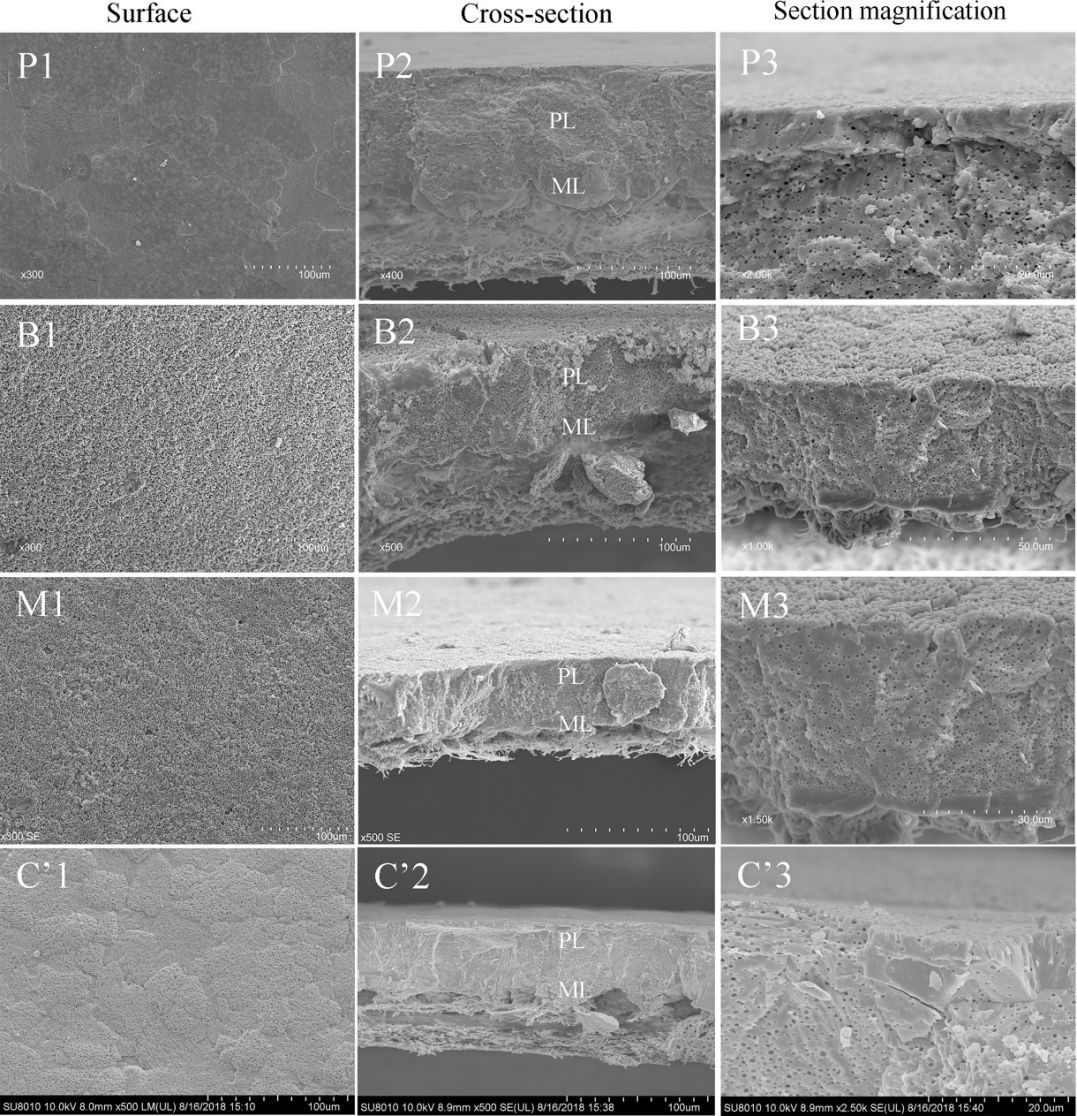
Eggshell surface and cross-section scanning electron micrographs of pigeon (P), budgerigar (B), munia (M), and canary (C’) eggs. The eggshell of pigeon, budgerigar, munia, and canary was divided into a mammillary layer (ML) and a palisade layer (PL). Budgerigar, munia, and canary eggs have many honeycomb vesicles in the organic matrix on the eggshell surface and in cross-section.

**Fig 3:**
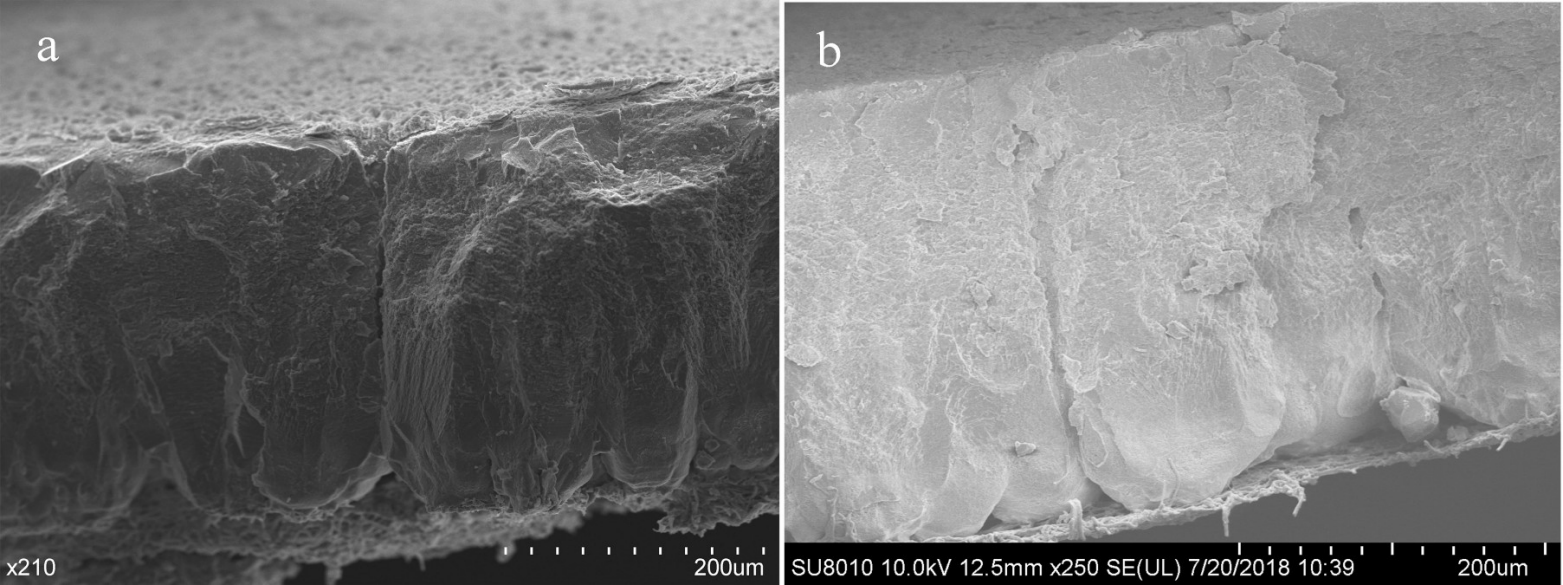
**(a) Exposed and (b) unexposed pores on the eggshell surface.** Pores are the pathway for vapor exchange and invasion of microbes. Cuticles cover the outer pore orifice and form a barrier against bacterial trans-shell penetration.

Shell cross-sections of chicken, turkey, quail, duck, goose, and ostrich eggs could be divided into mammillary, palisade, and cuticle layers (Fig 1), whereas pigeon, budgerigar, munia, and canary eggshells lacked the cuticle layer (Fig 2). Compared with eggs of other species, quail eggs had the thickest cuticle layer. Ostrich eggshells had thin and patchy cuticle layers. Quail and turkey eggs had much more tightly connected cuticle nanospheres, in contrast with chicken and goose eggs. Ostrich eggshells also had cuticle nanospheres at larger magnification. Interestingly, duck eggs seemed to have highly atypical cuticle nanospheres compared with the other species. The pores would be an opening for bacteria, if there were no cuticle coverage on the eggshell (Fig 3).

#### Eggshell pores

**Fig 4:**
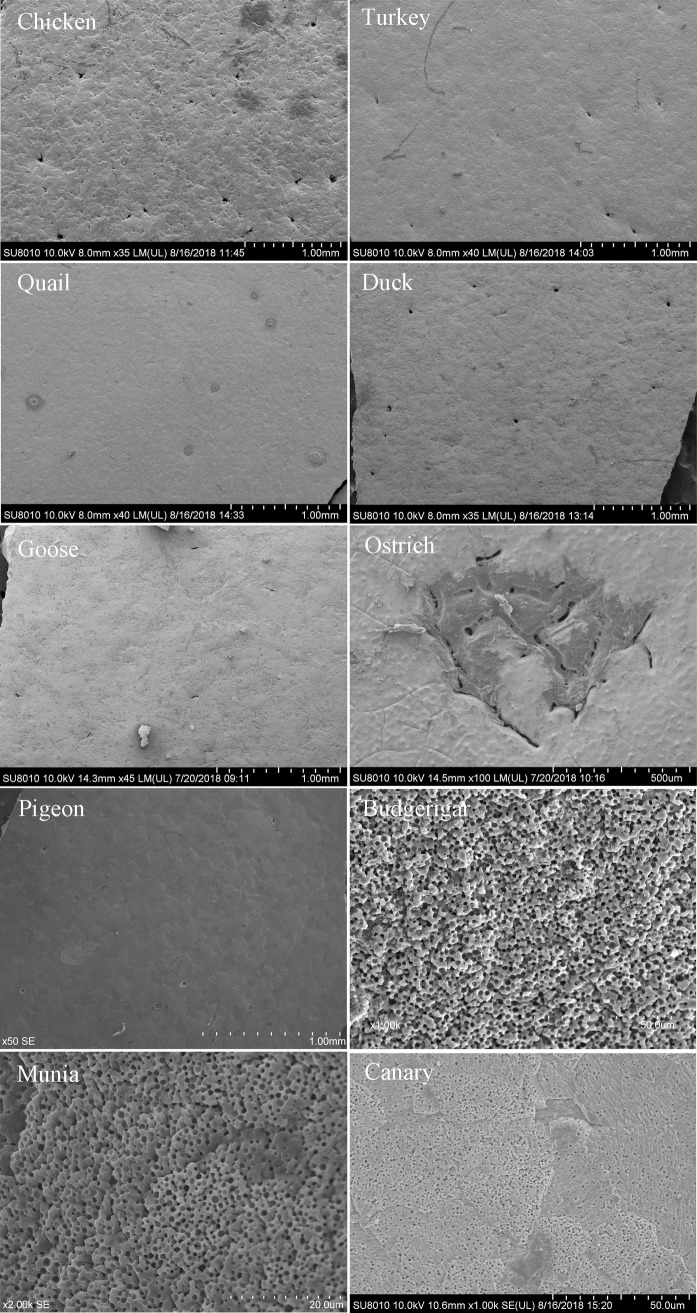
The pore characteristics on the eggshell surface of ten species in SEM. Pores on the outer surface of eggshells for chicken, turkey, quail, duck, and pigeon are near-circular, while those on goose eggshell are crescent-moon-shaped. Ostrich eggshell pores are considerably larger and more irregular than eggshell pores of other species. Budgerigar, munia, and canary eggshells have honeycomb-like pores.

Goose and ostrich eggs exhibited irregularly shaped pores, while eggs of the remaining tested species had nearly round pores (Fig 4). To make the results more precise and comprehensible, the pore diameter of different species was measured in different ways. Goose egg pores were somewhat like a “crescent moon”, therefore, goose eggs’ pore diameters were measured by the short axis. Ostrich eggs showed many irregular porosities; hence, the pore diameter of ostrich eggs was determined by the diameter of average pore diameter of each small pores and the sum of small pores’ areas was used as the single pore area. Duck eggs had the largest pore diameter (17.21 ± 5.48 μm). Budgerigar, munia, and canary eggs had honeycomb-like pores, and the pore diameter and pore area of those species’ eggs was significantly smaller (0.57 to 1.22 μm, *P* < 0.05) than that of the eggs of other species. Ostrich eggs had the lowest pore density (7.17 ± 215 per cm^2^) among all tested eggs, coupled with the largest pore area (23148.14 ± 9891.43 μm^2^). Goose eggs had a larger pore area than the chicken, turkey, quail, duck, and pigeon eggs.

#### Eggshell thickness

The ostrich egg had the thickset eggshell (1955.51 ± 93.99 μm), followed by goose eggs (486.94 ± 60.30 μm) (Table 1). The eggshell thickness of budgerigar, munia and canary eggs was much smaller (57.18 to 92.61 μm, *P* < 0.05). The eggshell of the quail egg was thicker than that of pigeon eggs (192.18 ± 19.32 μm and 195.77 ± 111.56 μm, respectively, *P* < 0.05).

#### Egg size

**Fig 5:**
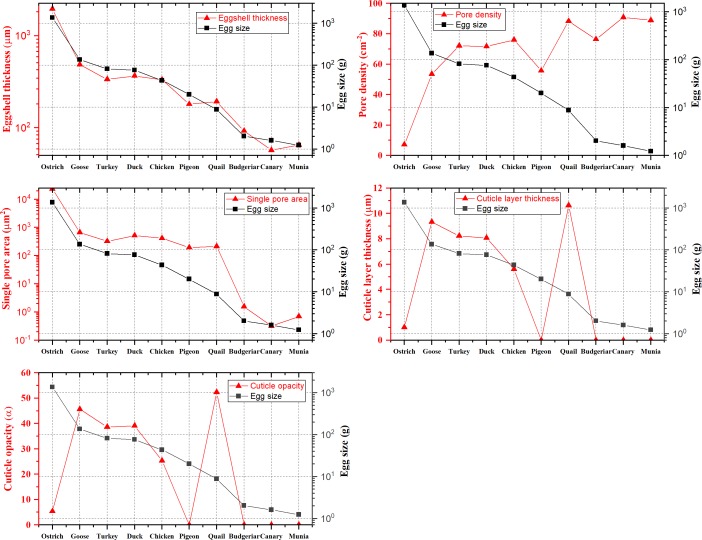
Variation of eggshell traits as a function of egg size. As egg weight, eggshell thickness, and pore area showed great variation among the eggs of the ten bird species, those traits are shown on a logarithmic scale. The egg size was determined by egg weight according to the results in Table 1. The egg weight decreases from ostrich to canary. Eggshell thickness and pore area decrease with egg size. Cuticle opacity, and cuticle layer thickness do no display a significant trend with egg size.

Eggs size was determined by egg weight as shown in Table 1. Ostrich eggs had the largest egg size (1357 ± 82.20 g), while budgerigar, munia, and canary egg weight ranged from 1.23 to 2.02 g, which was significantly smaller than that of the other eggs (*P* < 0.05). The eggshell thickness and pore area were positively correlated with egg size (Fig 5). The pore density seemed to decrease with increasing egg size. Cuticle opacity and cuticle layer thickness were not significantly correlated with egg size.

### *E*. *coli* penetration

**Fig 6:**
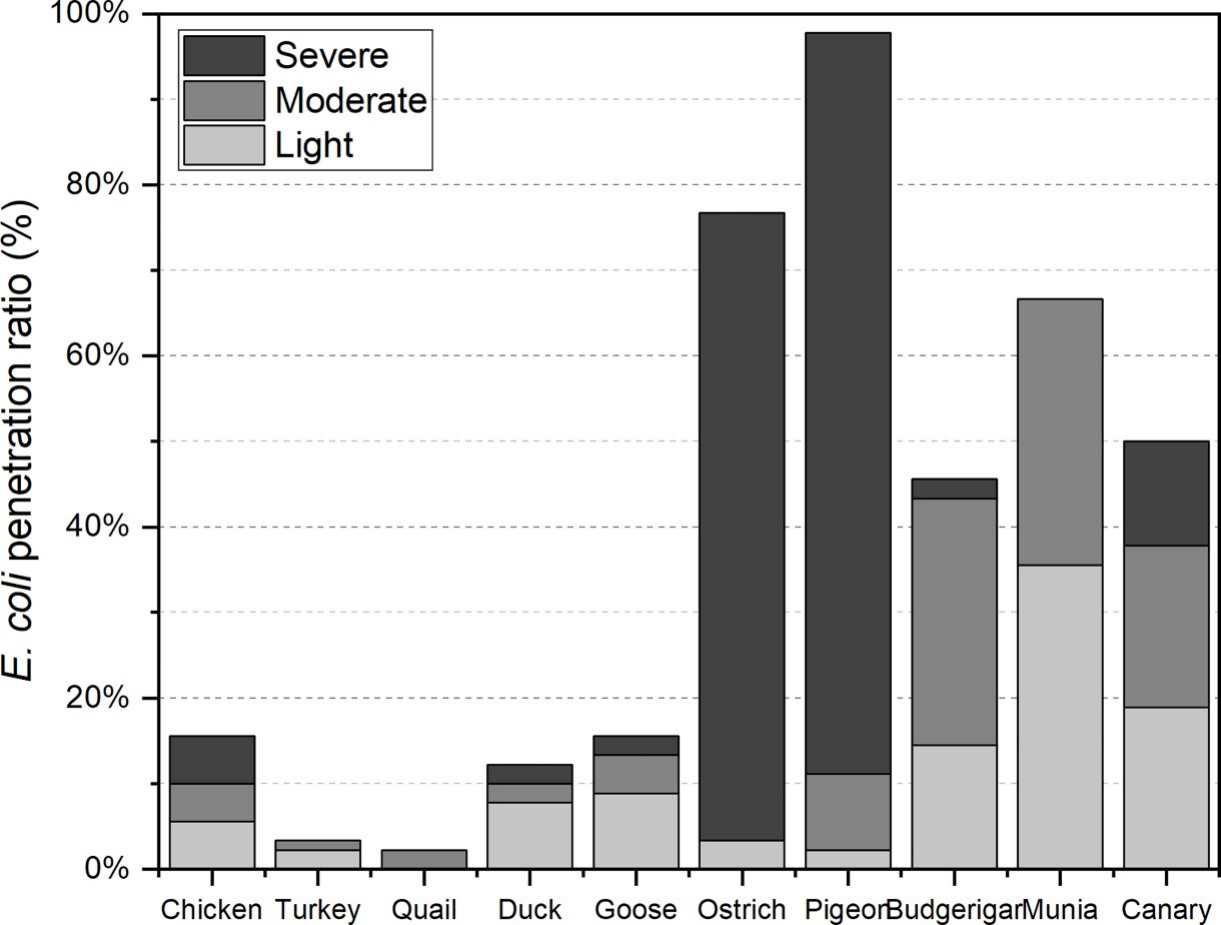
*E coli* contamination grades for the ten species. Light (light gray), moderate (gray), and severe (dark gray) contamination represent 1 to 3, 4 to 10, and more than 10 *E*. *coli* luminescence spots on the inner surface of one eggshell, respectively.

Among all the investigated species, the egg penetration rates for pigeon and ostrich were 97.78% and 76.67%, respectively, which was much higher than that for the other species (Table 1). Further, most pigeon eggs and ostrich eggs were severely contaminated by *E*. *coli* (Fig 6). Quail and turkey eggs had a lower contamination rate (2.22% and 3.33%, respectively), and these eggs were lightly and moderately contaminated. The penetration rates for munia, budgerigar, and canary eggs was 68.33%, 46.67%, and 50%, respectively.

### *E*. *coli* penetration and eggshell traits

**Fig 7:**
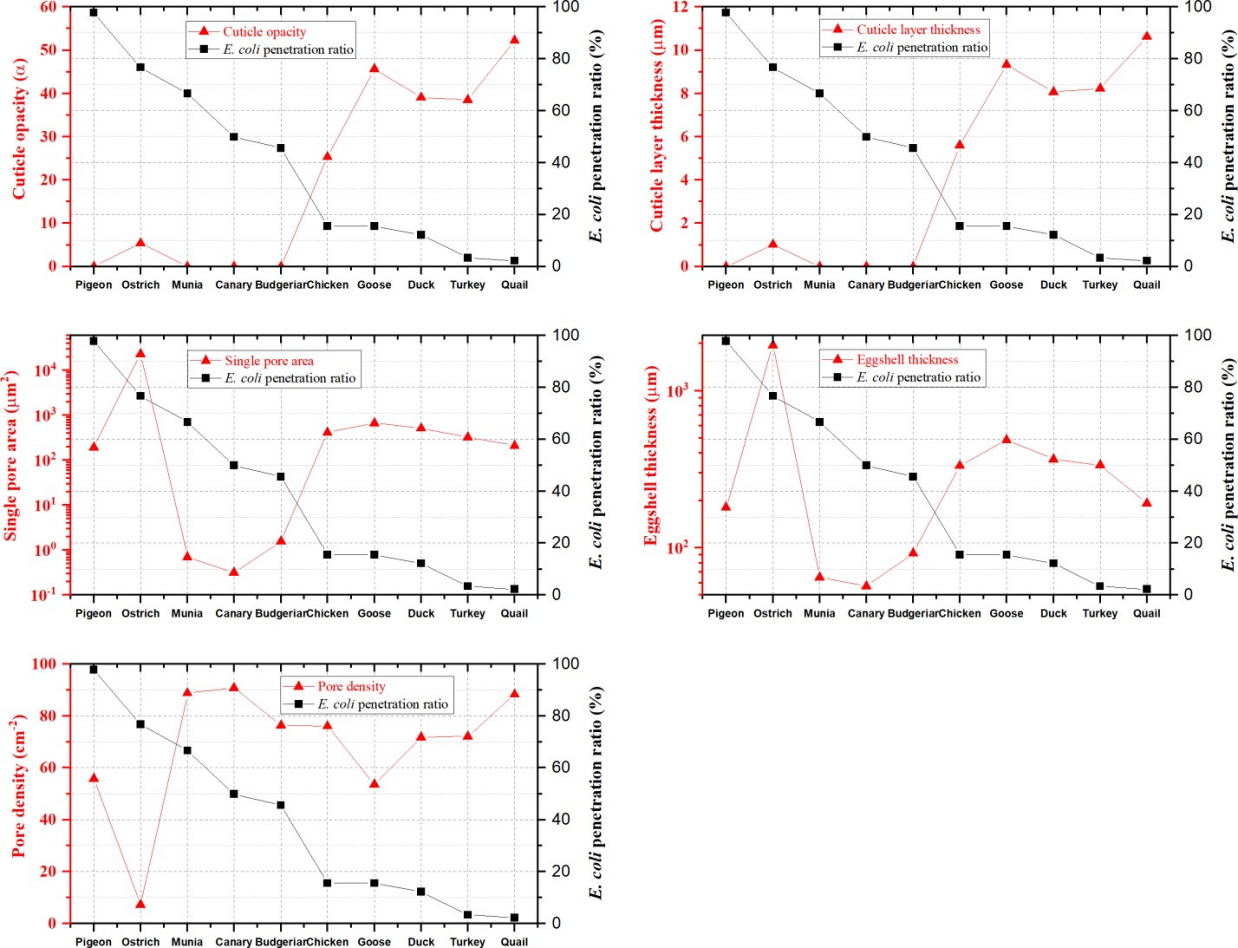
The influence of eggshell traits on *E*. *coli* penetration. Since eggshell thickness and pore area showed great variation among the ten bird species’ eggs, those traits are shown on a logarithmic scale. The *E*. *coli* penetration rate decreased from pigeon to quail.

The influence of eggshell traits on the antibacterial effectivity is shown in Fig 7. From Fig 7, it can be seen that eggs with a thick cuticle layer and high cuticle opacity tend to have a lower *E*. *coli* penetration ratio, proving that for birds, the cuticle is an effective barrier against bacterial penetration. As for the single pore area, pore density, and eggshell thickness, none of those exhibited a consistent trend with bacterial penetration among these ten bird species.

#### Precocial and altricial birds’ eggs

**Fig 8:**
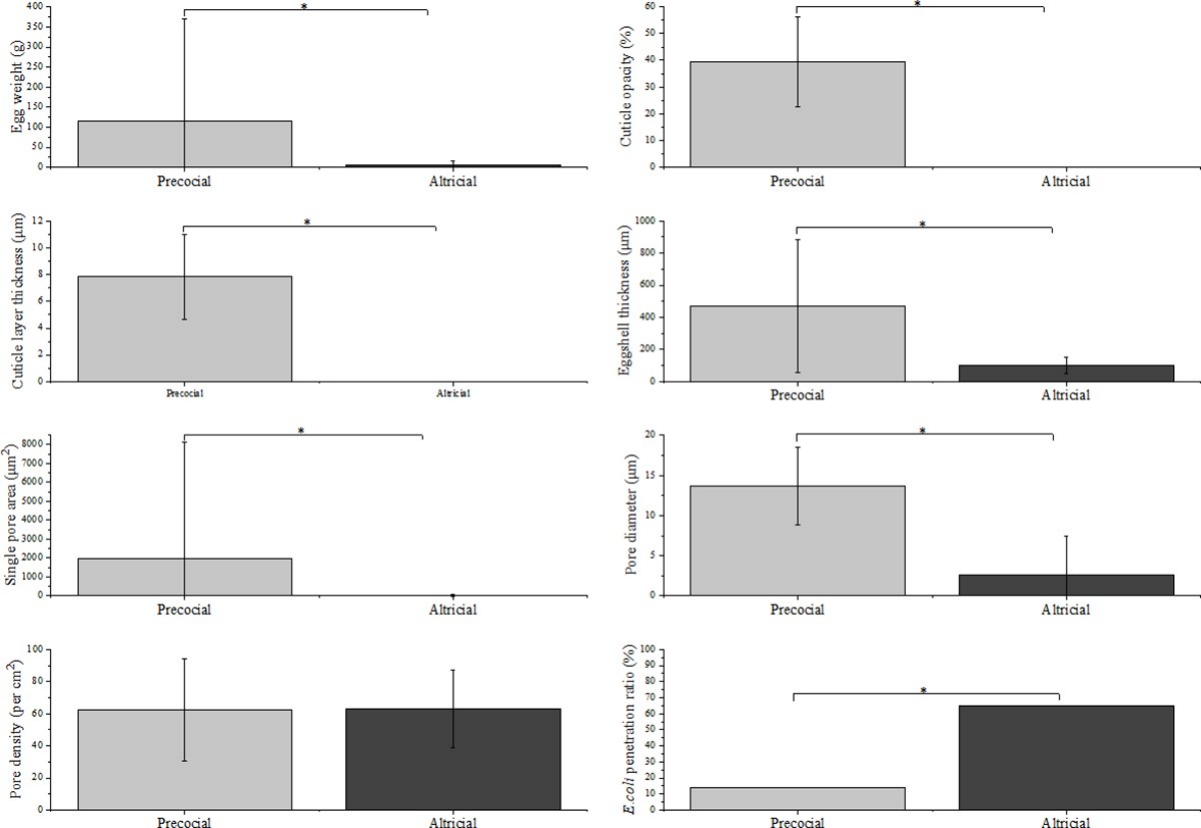
The difference in eggshell traits and *E*. *coli* penetration ratio between precocial and altricial birds’ eggs. A significant difference in eggshell traits and *E*. *coli* penetration ratio between precocial and altricial birds’ eggs is indicated by an asterisk (*).

At 65%, altricial birds’ eggs showed a much higher *E*. *coli* penetration rate than precocial birds’ eggs (13.96%) (*P* < 0.05) (Fig 8). Eggshell thickness, single pore area, and pore diameter of precocial birds’ eggs were significantly larger than those of altricial birds’ eggs (*P* < 0.05). There was no significant difference between precocial and altricial birds’ eggs for pore density (*P* > 0.05).

## Discussion

Chicken, turkey, quail, duck, and goose eggs with high cuticle opacity had much lower *E*. *coli* penetration rate than ostrich, pigeon, budgerigar, munia, and canary eggs with a poor or no cuticle layer (Table 1). It showed that eggshell cuticle can effectively defend against bacterial penetration [5,6,15,24]. Besides, from Fig 7, it can be predicted that if the cuticle opacity of a bird species’ eggs is higher than 25.34 or their cuticle layer is thicker than 5.6 μm, the eggs’ *E*. *coli* penetration rate would be lower than 15.56%. Quail and turkey eggs had a lower penetration rate, which may be due to the cover of tightly connected nanospheres on the eggshell [11]. It has also been reported that the cuticle of chicken had moderate heritability [6,25]. These results suggest that we can improve cuticle quality by breeding to enhance the antibacterial ability of eggshell.

Scanning electron micrographs indicated that precocial birds’ eggs (chicken, duck, goose, turkey, quail, and ostrich) had a cuticle layer on their eggshell surface, whereas altricial birds’ eggs (pigeon, budgerigar, munia, and canary) did not. It was reported that cuticle evolution was related to environmental pressure [6,8,26]. As we know, high ambient humidity is more conductive to microbial growth [27]. Birds nesting in habitats with higher infection risk (e.g., wetter and warmer) were more likely to evolve a cuticle layer on their eggshells than those nesting in lower-risk habitats [9,28]. Our results that goose and duck eggs, which are always laid in a swamp environment, have a thicker eggshell cuticle than chicken and ostrich eggs, which are usually laid in low ambient humidity, matched well with what has been reported previously.

Most altricial birds perch on trees, while most precocial birds nest on the ground. Moreover, altricial mothers, such as Passeriformes and Columbiformes, are generally so careful in hatching eggs that they use their feathers or the epidermal layer of their brood patches to inoculate shells with antibiotic agents, reducing the possibility of microbial infection [29,30,31,32], while precocial birds like Anseriformes always hatch in muddy places where the eggs are at high risk of being contaminated by pathogenic microorganism. According to the theory of evolution, after a long time of natural selection, the cuticle quality of altricial bird eggs seems to be getting worse and some even lack cuticle deposition on the eggshell surface. Certainly, this hypothesis needs to be verified with more eggs from different bird species along with an evolutionary genomics analysis.

Absence of a cuticle layer on the eggshell surface totally exposes the pores (Fig 3), which would increase the possibility of microbe contamination [7,18], leading to higher *E*. *coli* contamination rates in altricial bird eggs. Budgerigar, munia, canary, and pigeon eggs all lack cuticle deposition on the eggshell; however, eggs of the former three species had a far lower penetration ratio than pigeon eggs. The results can be explained by the relationship between pore diameter and average bacteria size. The average diameter of spherical bacteria is 0.5 to 2.0 μm [33,34], and rod-shaped or filamentous bacteria are 1–10 μm long, with diameters of 0.25 to 1.0 μm [35]. Pore diameters for budgerigar, munia, and canary ranged from 0.57 to 1.22 μm (Table 1). Bacteria have more difficulty in penetrating through the eggshell if the pore diameter is quite close to or even smaller than their size, implying low contamination. In other words, when the eggs’ pore diameter or single pore area is quite close to or even smaller than the size of bacteria, it could be an effective protection against bacterial trans-shell penetration. A large pore diameter on eggshell would make bacteria penetrate more easily. This is why goose eggs, with a large pore area, have a higher bacterial penetration ratio than quail and turkey eggs despite the high cuticle opacity of goose eggshell.

Ostrich eggs, with large pore diameter and large pore area, have a quite high contamination rate in this study. However, in natural habitats, the ostrich’s bacterial contamination risk is not so high [36]. This can be attributed to the low ambient humidity and high ambient temperature in the ostrich habitat, which is not conducive to bacterial reproduction [37,38]. Additionally, the thick ostrich eggshell (almost 2 mm) may be an effective physical barrier against trans-shell bacterial contamination [15,19]. A thick eggshell indicates a long trans-shell distance (Fig 3), which can increase the difficulty and penetration time for bacterial trans-shell invasion.

Pigeon eggs, which lack cuticle and have a large pore diameter, exhibited the largest *E*. *coli* penetration ratio among all tested eggs, and 86.67% of pigeon eggs were severely contaminated in this study. Egg antibacterial behavior is usually achieved through a very complex mechanism. Embryo protection is not only ensured in the first place by a physical barrier, the shell, but also by a complex system of chemical defenses including antibodies and a variety of antimicrobial proteins in egg albumen and the eggshell membrane. Our team’s previous research found that pigeon egg albumen contained higher concentrations of lysozyme C, ovalbumin, ovotransferrin, and heptoglobin than quail, turkey, duck and goose eggs [39]. Lysozyme, ovalbumin, and ovotransferrin play an important role in antimicrobial activity [40,41,42]. Heptoglobin, a natural bacteriostat [43,44], was found only in pigeon eggs, not in any other avian eggs [39].

In this study, the eggshell’s antibacterial effectivity in precocial and altricial birds was investigated. As discussed above, we can argue why altricial bird’s eggs have higher *E*. *coli* contamination rates than precocial bird’s eggs in this study. First, and most importantly, altricial birds’ eggs lack the protection of cuticle. Furthermore, the lack of cuticle leaves the pores of altricial birds’ eggs open on the eggshell surface, increasing the risk of trans-shell invasion by microbes. In addition, their thin eggshell might be another reason for the high contamination rate in altricial birds’ eggs.

## Conclusion

This comparative study measured the eggshell antibacterial effectivity in six precocial and four altricial birds’ eggs by focusing on morphological features (cuticle quality, pore density, pore diameter, and eggshell thickness). It was found that the cuticle can be an effective barrier against bacterial penetration. Precocial birds’ eggs with the protection of cuticle had lower *E*. *coli* penetration rates than altricial birds’ eggs with no cuticle layer on the eggshell. The evolution of cuticle may be related to the environment/selective pressure. Besides cuticle quality, the pore diameter plays an important role in the antimicrobial process of bird eggs as well. The results in this study provide insight into the eggshell antibacterial effectivity in precocial and altricial birds’ eggs, and can be a reference for antibacterial studies in eggs of various bird species in the future.
